# Changes in the genomic content of circulating *Bordetella pertussis *strains isolated from the Netherlands, Sweden, Japan and Australia: adaptive evolution or drift?

**DOI:** 10.1186/1471-2164-11-64

**Published:** 2010-01-26

**Authors:** Audrey J King, Tamara van Gorkom, Han GJ van der Heide, Abdolreza Advani, Saskia van der Lee

**Affiliations:** 1 Laboratory for Infectious Diseases and Screening (LIS) Centre for Infectious Disease Control, National Institute for Public Health and the Environment - RIVM - Netherlands, P.O. Box 1, 3720 BA Bilthoven, The Netherlands; 2 Swedish Institute for Infectious Disease Control, Nobels väg 18, S-171 82 Solna, Sweden

## Abstract

**Background:**

*Bordetella pertussis *is the causative agent of human whooping cough (pertussis) and is particularly severe in infants. Despite worldwide vaccinations, whooping cough remains a public health problem. A significant increase in the incidence of whooping cough has been observed in many countries since the 1990s. Several reasons for the re-emergence of this highly contagious disease have been suggested. A particularly intriguing possibility is based on evidence indicating that pathogen adaptation may play a role in this process. In an attempt to gain insight into the genomic make-up of *B. pertussis *over the last 60 years, we used an oligonucleotide DNA microarray to compare the genomic contents of a collection of 171 strains of *B. pertussis *isolates from different countries.

**Results:**

The CGH microarray analysis estimated the core genome of *B. pertussis*, to consist of 3,281 CDSs that are conserved among all *B. pertussis *strains, and represent 84.8% of all CDSs found in the 171 *B. pertussis *strains. A total of 64 regions of difference consisting of one or more contiguous CDSs were identified among the variable genes. CGH data also revealed that the genome size of *B. pertussis *strains is decreasing progressively over the past 60 years. Phylogenetic analysis of microarray data generated a minimum spanning tree that depicted the phylogenetic structure of the strains. *B. pertussis *strains with the same gene content were found in several different countries. However, geographic specificity of the *B. pertussis *strains was not observed. The gene content was determined to highly correlate with the *ptxP*-type of the strains.

**Conclusions:**

An overview of genomic contents of a large collection of isolates from different countries allowed us to derive a core genome and a phylogenetic structure of *B. pertussis*. Our results show that *B. pertussis *is a dynamic organism that continues to evolve.

## Background

*Bordetella pertussis *is the causative agent of whooping cough, a highly contagious disease of the respiratory tract in humans. *B. pertussis *and the closely related *B. parapertussis *have diverged independently by genome decay of a *B. bronchiseptica*-like ancestor [1,2]. The three species have much in common, including a high degree of sequence similarity in shared genes. However, they differ in several respects such as severity of disease and host range specificity [3-6].

Extensive immunization of children reduced the incidence of serious disease and mortality caused by *B. pertussis *[7]. However, globally, pertussis remains a leading cause of vaccine preventable hospitalizations and deaths in children [8,9]. In the last decade, a resurgence of pertussis, especially among adolescents and adults, has been observed in many industrialized countries with a high vaccination coverage [10-12]. Numerous studies have demonstrated that the *B. pertussis *population has changed since the 1950s. Waning immunity, in combination with pathogen adaptation, may have contributed to the continued circulation of *B. pertussis *strains [13-18].

In most industrialized countries, generalized vaccination with a whole cell pertussis vaccine (WCV) began between 1950 and 1960. However, in the late 1970s, concerns over vaccination side effects led to changes in the vaccine composition, vaccine uptake, or temporary exclusion of pertussis whole cell immunization in some countries [19,20]. In order to tackle this problem, the development of an acellullar pertussis vaccine (ACV) exhibiting fewer side effects started in the 1980s. Immunization schedules and the type of vaccine used began to differ in each country, leading to disparities in pertussis vaccination histories. In the Netherlands, pertussis vaccination, using a WCV produced by the Netherlands Vaccine Institute, was introduced in 1953. From 1975 to 1985 a lower vaccine dose was used, which most likely lead to an increase in the incidence of pertussis between 1985 to 1987 [21]. After restoring the vaccine to the normal dose, a lower incidence of pertussis was noted until 1996. However, since 1996, the pertussis incidence has increased. Furthermore, pertussis epidemics have been frequently observed every 3-5 years. In 2005, the Netherlands ceased using WCVs and switched to using an ACV for primary and booster vaccinations. In Sweden, a WCV was used from 1953 until 1978. From 1979 to 1996, pertussis vaccinations were not administered. Since 1996, ACVs are used for pertussis vaccination in Sweden [22,23]. The Japanese population received the WCV from 1949 until 1981, at which time an ACV was introduced [24,25]. In Australia, a WCV had been used until 1997. However, in the late 1970's, the vaccine was not administered as frequently due to the population's decrease in confidence of the vaccine. Since 1999, and continuing until today, an ACV is used [26]. Senegal began large scale pertussis vaccinations with a WCV in the late 1980s, but the vaccination coverage was low [27]. The pertussis vaccination coverage in Kenya has also been relatively low [28,29].

A large number of techniques have been used to type *B. pertussis*. Commonly used methods are pulsed field gel electrophoresis (PFGE), Multi-Locus Sequence Typing (MLST) and Multiple-Locus Variable number of Tandem Repeat Analysis (MLVA) [23,30-37]. These methods have generally been effective for grouping strains of *B. pertussis*. However, some of these methods are very laborious, or have little discriminatory potential to elucidate the phylogenetic relationships of the strains.

Microarray technology, combined with mathematical analysis to determine phylogeny, has provided a sensitive and robust method to examine the genetic relatedness of bacterial populations [38-41]. Comparative genomic hybridizations (CGH) using genome-wide DNA microarrays have proven useful in studies of intraspecies diversity for a number of bacterial species [39]. Also, several microarray-platforms have been applied to specifically address questions related to the genome of *B. pertussis *[1,42-45]. In a previous study, we have used microarray-based CGH to determine the genomic characteristics of the *B. pertussis *strain involved in the Dutch 1996-2004 epidemic [45].

In this study, we sought to analyze the changes of the *B. pertussis *population in the Netherlands and five other countries, with different vaccination histories between 1949 and 2008. To this end, we determined the gene content of *B. pertussis *strains by using microarray-based CGH. The overview of gene content in a large collection of global isolates obtained from the CGH data enabled us to define the core genome of the *B. pertussis *species, and to construct a phylogenetic structure of *B. pertussis*. Furthermore, it gives us a better understanding of the dynamics of the *B. pertussis *strains circulating in the immunized population. Insight into the polymorphism of *B. pertussis *and its capacity to adapt to population immunity is important in the understanding of pertussis epidemiology.

## Results

### Strain collection

In this study, we analyzed 171 *B. pertussis *isolates by microarray-based CGH in order to investigate the dynamics of *B. pertussis *gene content, and possibly reveal temporal and geographic differences. We used a collection of 171 strains from six different countries (the Netherlands, Sweden, Australia, Kenya, Senegal and Japan) isolated between 1949-2008. The collection was divided into several time periods, chosen around significant events in the pertussis vaccination history of a particular country (Table 1). No strains were available in the post ACV period in Australia or from the vaccine free period in Senegal. The majority of the strains are isolated from the Netherlands; these strains should represent a sufficient number of *B. pertussis *populations in the Netherlands. We selected all strains based on frequency dependent selection using MLST type or PFGE type to avoid a selection bias. The Dutch strains of *B. pertussis *were typed in previous studies for MLST [45] [Mooi, unpublished results], while the Swedish strains were typed by PFGE [46]. The strains that were not typed in previous studies were typed in this study (not shown).

**Table 1:** Vaccination history of different countries

Country	Period	Comment	Year of Isolation	Number of strains
Netherlands	NV	vaccine strains		2
Netherlands	N1	pre-vaccination period	1949-1953	11
Netherlands	N2	Early post vaccine WCV	1953-1964	5
Netherlands	N3	late post vaccine WCV	1965-1975	6
Netherlands	N4	vaccine dose reduction period	1976-1984	16
Netherlands	N5	post vaccine dose reduction period	1985-1992	15
Netherlands	N6	Epidemic	1993-2004	31
Netherlands	N7	Early post ACV	2005-2008	18
Sweden	Sw1	Post WCV	1953-1978	5
Sweden	Sw2	Vaccine free period	1979-1996	7
Sweden	Sw3	Post ACV	1997-2008	8
Japan	J1	Post WCV	1949-1981	1
Japan	J2	Post ACV	1982-2008	7
Australia	A1	Post WCV	1950-1998	15
Australia	A2	Post ACV	1999-2008	-
Senegal	Se1	Vaccine free period	until 1987	-
Senegal	Se2	Early post WCV	1988-2008	10
Kenya	K1	Vaccine free period	until 2008	13
		Reference strain, Tohama I		1

### Microarray

We used two different microarrays to analyze the gene content of the *B. pertussis *strains. Forty-two percent of the strains were analyzed by a previously validated *B. pertussis *oligonucleotide microarray based on the Tohama I genome [45], while the remaining 58% of the strains were analyzed by a new extended oligonucleotide microarray based on the *B. pertussis *sequences supplemented with the additional CDSs from *B. parapertussis *and *B. bronchiseptica *[47]. Nine (5%) strains were analyzed using both microarrays. In total, 3,751 probes were included in the *B. pertussis *microarray and 5,910 probes were used in the extended *Bordetella *microarray (Additional file 1, Table S1a and Additional file 2, Table S1b). The efficacy of the extended *Bordetella *microarray was assessed in this study by the control hybridizations of reference DNA (genomic DNA from ATCC BAA-589 Tohama I) vs. reference DNA. The reference strain ATCC BAA-589 Tohama I did not hybridize to all probes on the microarray, since the array contains probes that are not present in this strain. The majority of the genes (99.83%) in this strain were predicted to be present/conserved (data not shown). The nine strains tested on both microarrays gave similar predictions regarding the absence/divergence and presence/convergence of the genes that were observed in both microarrays. We confirmed the absence of the genes predicted to be absent/divergent by targeted PCR assays (see Materials & Methods).

### Gene content, core genome and variable genes

We have applied microarray-based CGH to 171 *B. pertussis *strains in order to predict the gene content for these strains (Additional file 3, Table S2). In order to gain insight into which genes found previously in Bp/Bpp and Bb are present in the Bp species, we counted all of the different CDSs found in the 171 analyzed strains. The total number of CDSs predicted to be present in the isolates was 3,871, (i.e. the pan-*B. pertussis *genome consists of 3,871 genes). However, the fully sequenced and annotated *B. pertussis *strain (Tohama I) was determined to have 3,816 CDSs, a difference of 55 CDSs. These extra genes are clustered in eight regions of difference (RDs) (Table 2). Five of these RDs had previously been found in *B. pertussis *strains [48]. Here, 12 new extra genes, forming three RDs, were identified in *B. pertussis *strains; BPP0474/BB0474, BPP0511-12/BB0516-17 and BPP2338-47/BB1789-98 are also present in *B. parapertussis *strain 12822 and *B. bronchiseptica *strain RB50. Since the genes that are not present in the Tohama I genome were not observed in the *B. pertussis *microarray, all of the strains analyzed with this array were also screened by PCR for the eight RDs. In 99% of the strains, the genes BPP0529-36, BPP0931-50, BPP4293-4301 and BPP0474 were present (Table 2). BPP0822-24 and BPP0825-27 were present in 85% or 86% of all strains tested, respectively. The genes BPP2338-47 and BPP0511-12 were found in only 6% of the *B. pertussis *strains (Table 2).

**Table 2:** Extra genes not found in Tohama I genome

BPP and BB-number	No. of genes	Size (kb)	Percentage of presence
BPP0474/BB0474	1	1	99%
BPP0511-12/BB0516-17	2	2	6%
BPP0529-36/BB0534-41	8	10.5	99%
BPP0822-24/BB0916-8	3	7.4	85%
BPP0825-27/BB0919-21	3	3.3	86%
BPP0931-950/BB1141-59	20	17	99%
BPP2338-47/BB1789-98	10	13.8	6%
BPP4293-4301/BB4880-88	9	9.1	99%

**Figure 1:**
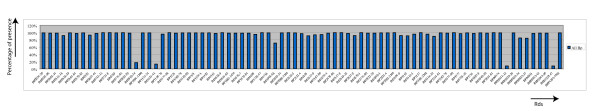
**The frequency at which regions of difference (RD) are present in *B. pertussis *clinical strains isolated in 6 different countries between 1949 and 2008**. Histogram of the average gene content of all 171 *B. pertussis *strains analyzed. Bars indicate the presence percentage of the gene or geneclusters (RDs).

**Figure 2:**
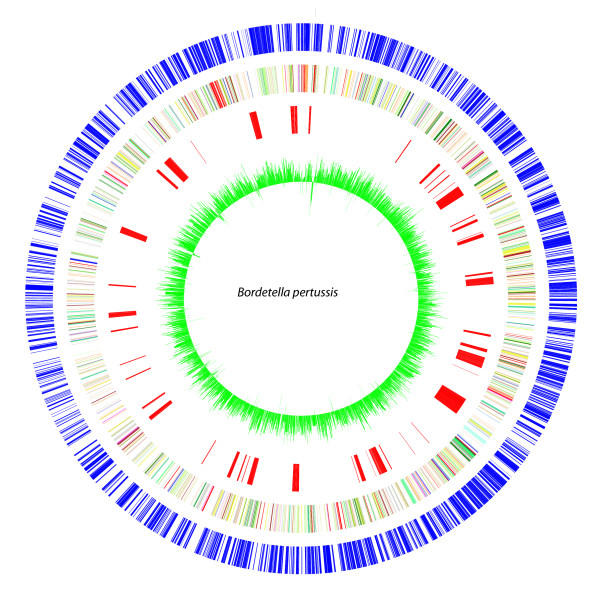
**The variable genes identified with microarray in this study, mapped onto circular *B. pertussis *genome Tohama I**. Variable genes not present in the Tohama I genome are not mapped in this figure. From the outside in, the outer circle 1 (blue) shows the position of the genes on the + strand. Circle 2 shows the COG functional categories on the + strand. The COG domains are color coded according to their main functional categories (for color code see additional file 8, Table S9). Circle 3 (red) indicates the position of the variable genes on the Tohama I genome. Circle 4 shows the GC % percentage of the Tohama I sequence.

Of the 3,871 genes of the *B. pertussis *pan-genome, 513 genes (13.3%) were detected as absent/divergent in one or more of the tested isolates, and were considered variable genes (Additional file 4, Table S3). The variable (non-core) genes formed a total of 64 RDs (Additional file 5, Table S4), each of them consisting of one or more contiguous CDSs. Sixty-seven percent of the 64 RDs were confirmed to be absent by targeted PCR assays or were also described by others (see Materials & Methods) [42,44]. Our results demonstrated that most variable genes were present in more than 80% of the analyzed strains, and only 43 of the non-core genes were absent frequently and present in less than 20% of the strains (Figure 1). These genes are BP0910-34, BP1135-41, BPP0511-12, and BPP2339-47. Besides the variable genes found in this study, previous studies have suggested that 76 additional genes are missing in at least one strain [42,44] (Additional file 6, Table S5). Based on our studies and reports by others, a total of 589 variable genes have been described for *B. pertussis*. Thus, 15.2% of the pan-genome is variable, while 3,282 genes (84.8% from pan-genome) are shared among all analyzed strains, and form the core genome of *B. pertussis *(Additional file 7, Table S6). The distribution of core and variable genes in the *B. pertussis *Tohama I genome is illustrated in Figure 2 (see also Additional file 8).

### Identification of Clusters of Orthologous Groups (COG) enriched for variable genes

**Figure 3:**
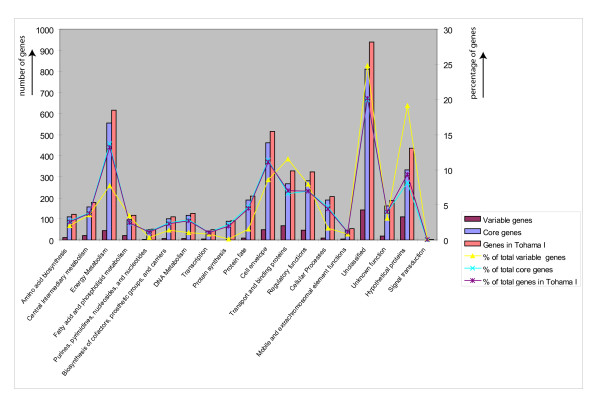
**Functional categories of core and variable genes in *B. pertussis***. The height of each bar represents the number of variable (purple), core (blue) and genes present in Tohama I (orange) indicated in specific gene categories. The lines indicate the percentages of the total, variable genes (yellow), core genes (blue) or genes in Tohama I (purple) in each category.

The proportions of the variable genes with respect to their functional category are shown in Figure 3. Genes involved in housekeeping functions (2,185 in total), such as small molecule metabolism and macromolecule metabolism, were found to be relatively conserved; variable genes accounted for only 8%. In contrast, the variable genes accounted for 16% of the total genes in the other 8 categories (i.e. transport and binding proteins, regulatory functions, cellular processes, mobile and extra chromosomal element functions, hypothetical proteins, signal transduction, unclassified and unknown function). In particular, genes involved in transport and binding, hypothetical genes and those encoding unclassified functions are overrepresented in the variable genes by 11%, 19% and 25%, respectively. Core genes are overrepresented in the genes involved in energy metabolism and most of the genes involved in macromolecule metabolism (Figure 3). Variable genes are enriched for pseudo genes, since we found that 12.5% of the missing genes are pseudo genes, while 9.6% of the Tohama I genes are pseudo genes.

### Lineage relationships revealed by phylogenetic analysis

**Figure 4:**
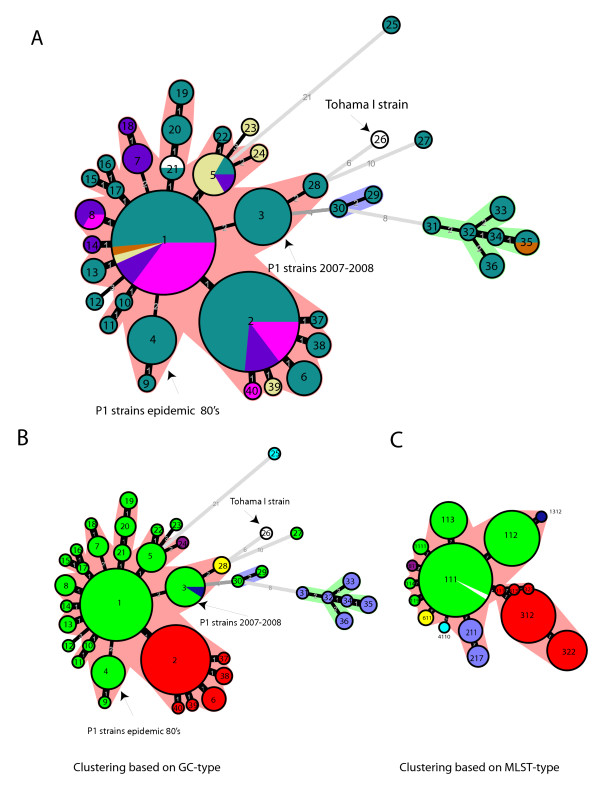
**Minimum spanning tree of 148 *B. pertussis *strains**. **A**. Each circle (node) represents a different GC-type, indicated by the number in the middle of the circle. The size of the circle corresponds to the number of strains within the particular GC-type. The number along the edge reflects the phylogenic distance between each neighboring node. A thicker edge corresponds to a shorter phylogenic distance. Color is based on country of isolation: Green = Netherlands, Pink = Sweden, Yellow = Japan, Purple = Australia, Brown = Dutch vaccine strains 509 and 134, White = Tohama I strain, Wellcome 28 strain. **B**. Minimum spanning tree as in Figure 4A, color based on *ptxP *type. Green = *ptxP1*, Blue = *ptxP2*, Red = *ptxP3*, Turquoise = *ptxP4*, Yellow = *ptxP6*, Purple = *ptxP8 *and Darkblue = *ptxP13*, White = Tohama I strain. **C**. Minimum spanning tree based on MLST-types, MLST designation was based on the allele number in the order *ptxP*, *fim3 *and prn region 1 (e.g. MLST 113 represents strains with *ptxP1*, *fim3-1 *and *prn3*). Each circle represents a different MLST-type, indicated by the number in the middle of the circle. The number along the edge reflects the phylogenic distance between each neighboring node. A thicker edge corresponds to a shorter phylogenic distance. Color code is as described in Figure 4B.

Next, we investigated the geographic and temporal differences in *B. pertussis *strains analyzed in this study. In order to be able to perform cluster analysis on the CGH data, RDs were categorized as either present (1), or absent (0) in each strain (Additional file 3, Table S2). In order to evaluate the relationships among *B. pertussis *strains, we performed a clustering analysis on the binary CGH data by the unweighted-pair group method using average linkages (UPGMA). This analysis generated a similarity matrix, as well as a UPGMA tree. Based on the similarity matrix, a minimum spanning tree was constructed to give a phylogenetic structure of 148 strains (Figure 4A and 4B). Based on the minimum spanning tree, 148 strains formed 40 different gene content types (GC-types) that were assigned into one large complex and two very small complexes.

**Figure 5:**
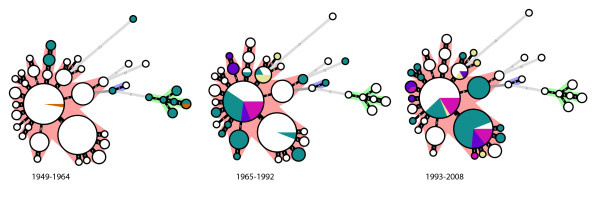
**Gradual change in gene content in *B. pertussis *strains**. Minimum spanning tree as shown in Figure 4A, but only strains isolated in indicated time period are colored. Color code is as described in Figure 4A.

We compared the frequencies of GC-types in three periods: 1949-1964, 1965-1992 and 1993-2008 (Table 3) (Figure 5). The most common GC-types are GC-type 1 (25% of the strains) and GC-type 2 (23% of the strains). The remaining GC-types found at frequencies lower than 8% were pooled together. Almost all (84%) of the strains analyzed from the first period were Dutch strains, because only a few strains from this period were available. In the 1^st ^period a dominant GC-type was not found, while 11 different GC-types were detected at a frequency of 5-11%. In the 2^nd ^period, strains carrying GC-type 1 increased in frequency to 41%, however, the frequency of these strains decreased again to 19% in the 3^rd ^period. Strains of GC-type 2 were detected at a frequency of only 5% in the 2^nd ^period, but were predominant (43%) in the 3^rd ^period. The differences observed in GC-type frequencies of different countries may be influenced by many factors, such as differences in vaccine history or other geographic factors. Nevertheless, we observed that strains carrying the same, or similar, gene content were found in different countries within the same time-period, and have seen no clear geographic specificity of the *B. pertussis *strains. For example, strains with the GC-type 1 and GC-type 2 were found in 4 and 3 different countries, respectively. Due to differences in vaccine history, specific lineages can arise in a country. For example, in the Netherlands, we observed an increase in strains with GC-type 4, which was the result of a change in the vaccine dose, and ultimately led to the 1980s pertussis epidemic. However, strains with this gene content were not found in other countries.

**Table 3:** Frequencies of GC-types during 1949-964, 1965-1992 and 1993-2008

	1949-1964				***1965-1992***				1993-2008			
**Country**	****GC-type 1****	****GC-type 2****	****Other GC-types****	****N****	***GC-type 1***	***GC-type 2***	***Other GC-types***	***N***	**GC-type 1**	**GC-type 2**	**Other GC-types**	**N**
												
The Netherlands	**0**	**0**	**100**	**16**	*32*	*5*	*62*	*37*	14	47	39	49
Sweden	**0**	**0**	**0**	**0**	*100*	*0*	*0*	*7*	46	38	15	13
Australia	**0**	**0**	**0**	**0**	*43*	*0*	*57*	*7*	0	50	50	8
Japan	**0**	**0**	**0**	**0**	*0*	*0*	*100*	*3*	20	0	80	5
Other	**33**	**0**	**67**	**3**	*0*	*0*	*0*	*0*	0	0	0	0

Total	**5**	**0**	**95**	**19**	*41*	*4*	*55*	*54*	19	43	39	75

GC-type frequencies are given in percentages

38 GC-types are found in low frequencies: GC-types 3-40.

**Figure 6:**
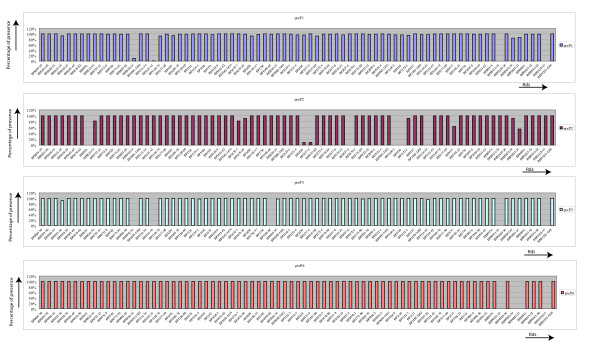
**Histogram of the average gene content of strains carrying *ptxP1*, *ptxP2*, *ptxP3 *and *ptxP6 *types**. Bars indicate the presence of the gene or geneclusters in all strains analyzed with a particular *ptxP *type, *ptxP1 *(n = 91), *ptxP2 *(n = 9), *ptxP3*(n = 39) and *ptxP6 *(n = 9).

In order to determine if the gene content was linked to specific alleles, we combined the gene content data with the MLST (*PtxP*-*Fim3*-*Prnregion1*) data. The gene content of *B. pertussis *strains was found to correlate highly with the *ptxP *type of the analyzed strains (Figure 4B). Moreover, clustering based on MLST-type (*PtxP*-*Fim3*-*Prnregion1*) data resulted in a similar structure (Figure 4C) compared to clustering based on GC-type data (Figure 4B). In our previous study, we found that all Dutch strains carrying the *ptxP3 *allele were characterized by the absence of the BP1948-66 gene cluster [45]. Here, we confirm the association of the *ptxP3 *allele with the absence of these genes also found in strains isolated from Sweden, Australia and Japan (n total= 39). Figure 6 shows the average gene content of all analyzed strains analyzed (n = 171) and is organized by *ptxP *type. The *ptxP3 *strains also miss BP0910-34 and BP1135-41, BPP2338-47 and BPP0511-2 besides the already mentioned RD: BP1948-66 (Figure 6). Furthermore, we also found that *ptxP2 *strains (n = 9) are characterized by the absence of BP0593, BP2627-9, BP3104-10, BP3314-22, and BPP0474 (Figure 6). *ptxP6 *(n = 9) strains are characterized by the presence of all RDs except for, BPP0822-4, BPP0825-7, BPP2338-47 and BPP0511-2 (Figure 6). Finally, the *ptxP1 *strains (n = 91) miss BPP2338-47 and BPP0511-2, as well as BP0910-34 and BP1135-41 in most of the ptxP1 strains. The *ptxP1 *strains are more diverse in gene content as several lineages were identified. A lineage of *ptxP1 *strains expanded in the 1980's is characterized by the absence of BP1698 and BP2167-80 (Figure 4B). A second *ptxP1 *clone was detected in the Netherlands after the year 2000, and is characterized by the absence of BPP0822-4 and BPP0825-7. A single strain isolated from the Netherlands in the year 2008 carries the *ptxP13 *allele and also has the same gene content (Figure 4B).

### Estimation of genome size

**Figure 7:**
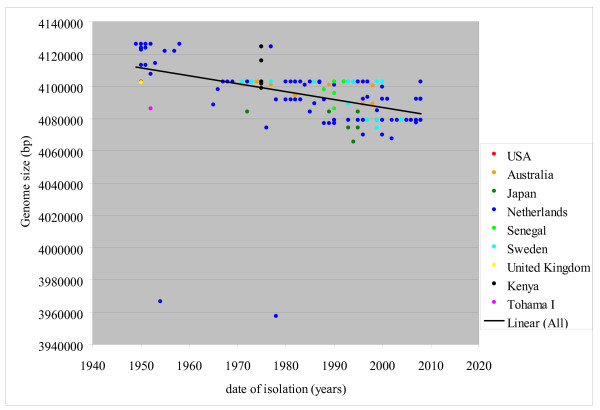
**Decrease in genome size in time**. Each dot stands for the calculated approximate genome-size of each strain plotted against the isolation year (n = 171). The color of the dots is based on the country of isolation. Strains with the same genome size and isolated in the same isolation year will be visible as one dot.

We calculated an approximate genome size for each strain based on the CGH data. The total number of genes per genome varied between 3,851 and 3,684 genes. The genome size of the *B. pertussis *Tohama I strain (4,086,186 bp) [47] was used as the starting point. We added the gene size for additional genes and subtracted the gene size for genes scored as being absent in a particular strain. Since microarrays can only detect genes present on the microarray, we estimated the genome size of these strains based on the absence/presence of the 3,871 genes of the *B. pertussis *pan-genome only. Figure 7 shows the estimated genome sizes for all 171 strains organized by isolation date. Remarkably, the genome size of *B. pertussis *strains appears to be significantly decreasing progressively (P < 0.001). A decrease in genome size, over time, was observed in strains isolated from the Netherlands, Sweden, Japan and Australia. However, we could not detect a decrease in genome size of Kenya and Senegal strains since strains from different isolation dates were not available. Strains with the same estimated genome size were found in different countries. These strains carried the same gene content (GC type), but different MLST types (Additional file 3, Table S2) which suggested that they did not simply arise from clonal expansion. Two major outliers exhibiting a smaller genome were also found in the Netherlands.

The largest genome size is estimated to be 4,124,398 bp (strains B0558 and B0572 isolated respectively in 1949 and 1952) and the smallest is 3,937,304 bp (strain B0295 isolated in 1978). The maximum size difference is 187,094 bp. The core genome (3,282 genes) is theoretically the smallest genome with which *B. pertussis *can survive. Based on the size of the present genes, the size of the core genome is estimated to be 3,485,846 bp.

## Discussion

The main purpose of the present study was to analyze the changes in gene content of the *B. pertussis *population in the Netherlands and five other countries between 1949 and 2008. This analysis was conducted using microarray-based CGH methods in order to gain insight into the dynamics of the *B. pertussis *population. Microarray-based CGH has been widely used to assess the genome variability among bacterial species or closely related bacteria. Given that sequencing of strains on a large scale is still time-consuming and laborious, CGH may resolve the problem to some extent by applying the available genome sequence information. This method can supply additional information about the genome composition and also provide the opportunity to analyze many more unsequenced strains on a genomic scale. Even though this technique has several limitations, we believe that the use of CGH technology, combined with confirmatory PCRs, allows sufficient assessment of the genetic diversity and gene content among *B. pertussis *strains. The high-throughput capability of microarrays enabled us to analyze more than 170 strains. One limitation of microarray-based analysis is that the detection of elements is limited to the presence of a gene in the microarray. In these studies, we used a microarray that is based on the sequence of the Tohama I *B. pertussis *strain, as well as all of the extra genes found in *Bpp *strain 12822 and *Bb *strain RB50. The Tohama I strain was shown not to be a good representative for *B. pertussis *strains [49]. Full genome DNA sequencing of three other *B. pertussis *strains by Bouchez *et al *[48] showed that not all of the novel genes were found in the Tohama I strain, but originated from the *Bpp *or *Bb *species. Since *B. pertussis *does not appear to acquire any new genetic material [1,42,49], we expect that if novel genes are present in the *B. pertussis *strains analyzed in these studies,they are likely to originate from *Bpp *or *Bb *species. By using a *Bordetella *pan-microarray, most of the genes present in the *B. pertussis *strains will be covered. Future DNA sequencing of additional *B. pertussis *strains will demonstrate if this assumption is justified. In this study, indeed the analysis of 171 *B. pertussis *strains identified the presence of 12 new genes in the *B. pertussis *species, which originate from the *B. parapertussis/B. bronchiseptica *species.

CGH analysis for a large series *B. pertussis *strains, isolated in six different countries, has resulted in an estimate of the core genome composition of *B. pertussis. *This analysis has also suggested the degree and nature of the genome flexibility between strains. A total of 3,282 genes were identified as belonging to the core genome. These genes were found in every strain analyzed until present time (this study and [42-45]), and appear to be essential to the lifestyle of this bacterium. The accessory (variably present) portion (589 genes) of the *B. pertussis *genome corresponds to about 15% of the pan-*B. pertussis *genome. Genes lost, assumed to not to be essential for *B. pertussis *survival in the human host, are enriched for genes involved in transport and binding, hypothetical genes and genes encoding unclassified functions. Accessory genes were confined to certain regions (RDs) in the chromosome, mostly flanked to an IS element (ISE) on at least one side of the RD. The IS 481 element, which is abundantly present (238 times) in the *B. pertussis *genome of the Tohama I strain, may be involved in the process of gene loss by facilitating homologue recombination between these perfect repeats within the genome. Previous studies have shown that repeats can be promoters of gene deletion [50,51], or can also result in large scale chromosomal rearrangements leading to disruption of the ancestral gene order [1].

Three main forces have been found to shape genome evolution; gene gain, gene loss and gene change [52]. Gene loss seems to be an important event in the genomic evolution of the *B. pertussis *species. *B. pertussis *evolved from a *B. bronchiseptica*-likeancestor probably by large scale gene loss [47]. Much of this loss is most likely due to ISE-mediated deletion events and/or ISE mediated rearrangements, which reshaped the genome presumably to benefit from increased virulence expression. In evolution from *B. bronchiseptica *to *B. pertussis *the genes that are lost or inactivated are generally those involved in membrane transport, small molecule metabolism, regulation of gene expression and synthesis of surface structures [47]. It seems that gene loss began during the evolution from *B. bronchiseptica*, and is continuing during the evolution of *B. pertussis. *This conclusion is supported by the results presented in this study that illustrate a progressive decrease in the genome size over time in strains isolated in different countries. Moreover, the genes that were lost exhibited similar functions as the genes lost in the evolution from *B. bronchiseptica *to *B. pertussis *(see above). Gene loss in *B. pertussis *has been previously reported for Finnish [44] and French *B. pertussis *strains [48]. However, to our knowledge, such a rapid decline in gene loss during a period of 60 years has not yet been described. The loss of genetic material is a dynamic, ongoing process that is not specific for one country, but observed in several areas of the world. In other examples of bacteria gene loss, the process has been described as a progressive purging of unnecessary genes from the genome [53]. Bacteria appear to prefer gene deletions, which could account for a general drive to lose DNA. It is generally assumed, that the bacterial deletion which offers the least negative fitness effect on the host will be selected. However, gene loss can lead to benefits for the pathogen as well, because some gene products are detrimental to pathogenic lifestyle. For example, loss of the cadA gene from the Shigella bacterium has been correlated with an increase of Shigella pathogenicity [54]. Additionally, the preferential loss of bacterial cell surface determinants has been shown to result in an increase in virulence by reducing the number of targets that could be recognized by the human immune system [55].

An intriguing question is what other factors influence the size and content of bacterial genomes? The introduction of vaccination against pertussis has been associated with changes in the *B. pertussis *population [12,36]. Thus, we investigated whether pertussis vaccination, during several decades, could influence the size and genomic content of the *B. pertussis *population. Therefore, *B. pertussis *strains from countries with different pertussis vaccination histories were analyzed. Our collection of *B. pertussis *isolates were divided into time periods of significant events that were observed in the epidemiology of pertussis. In order to gain more insight into how the *B. pertussis *strains are related, we used the genomic content overview of the *B. pertussis *isolates to construct a phylogenetic structure. Recently, this method was also used, in a similar manner, to decipher the microevolution of *V. parahaemolyticus *[40].

Clustering based on genomic content showed that *B. pertussis *strains isolated in different countries are mostly similar and did not reveal a particular geographic region of a specific strain. However, an analysis of the gene content of *B. pertussis *strains over time demonstrated a gradual change in gene content over a period of 60 years (Figure 5 and Table 3). In the Netherlands, strains that were found approximately 60 years ago cannot usually be isolated today. In the last 15 years, strains with a different gene content (GC-type 2) have emerged in several countries. The increase of these strains has most likely caused or influenced the resurgence of pertussis in many countries in the last decade [10-12]. In general, strains have changed in the Netherlands, Sweden and Japan, somewhat irrespective of any difference in vaccination history. It is remarkable that the gene content of strains isolated during certain timeframes is similar for all countries, even in the countries with low vaccination coverage. These results suggest that the differences in vaccination history have little or no influence in this process.

On the other hand, in the Dutch *B. pertussis *population we have seen changes that seem to be influenced by alterations in herd immunity. For example, GC-types 31-36 strains, similar to the vaccine 509-strain, appear to be completely removed from the population following the commencement of vaccination. In contrast, strains with similar gene content to the other vaccine strain 134 (e.g. GC-type 1), have persisted until at least 2008. Additionally, shortly after a change in the vaccine dose in the Netherlands, we have detected the expansion of a clone of *B. pertussis *strains, characterized by the absence of BP1698 and BP2167-80, and identified as GC-types 4 and 9. This clone, also characterized by MLVA types 130, 132, 134 and 137 [21] was able to expand between 1978 and 1988, leading to a pertussis epidemic between 1983 and 1988. However, this clone disappeared again following restoration of the vaccine dose to the original level. Herd immunity was possibly lost because of a decline in vaccine-derived immunity, which was a result of a change in the vaccine composition. This deviation appears to have selected for a change in the composition of circulating *B. pertussis *strains. In 2007-2008, we found the expansion of another clone of *B. pertussis *strains with an additional deletion (e.g. BPP0822-27). It is not yet known what factor induced the expansion of these strains, although the introduction of the ACV in 2005 in the Netherlands may be involved. However, we detected a single strain with the same gene content for the first time in 1996. In Sweden, where vaccination was ceased for a longer period, we did not see the rise of new *B. pertussis *clones that were involved in epidemics. This may possibly be due to the low number of isolates tested. During the last 15 years, the expansion of *B. pertussis *strains in multiple countries that carry the *ptxP3 *allele [18,45], may also have been influenced by intensive pertussis vaccination for half a century. Since pertussis has shifted to older age groups [11,56] in immunized populations during the last decade, it has been suggested that *B. pertussis *has adapted to the host population with waning immunity, in order to maintain the bacterial reservoir among older hosts [18,57]. Mooi *et al *[18] proposed that strains surviving better in these hosts are being selected for by vaccination of young infants. Recently, it was shown that the rise of strains carrying the *ptxP3 *allele, which are also characterized by a particular gene content [45] (GC-types 2, and its derivatives) and seem to have a benefit in older hosts, has contributed to the resurgence of pertussis in the Netherlands [18]. Thus, the size and gene content of the *B. pertussis *genome appear to be influenced by vaccination by herd immunity. Immune pressure may select for certain strains with a particular advantage, and which may be linked to specific gene content. The fact that we see the same or similar strains in different countries during certain time periods suggests that an important advantage for these strains may be their capability to spread throughout the immunized population. Importantly, strains with the same GC-type were found to possess different MLST types, which suggest that they are not a single clone.

In our previous study we found that all Dutch strains carrying the *ptxP3 *allele were characterized by the absence of the BP1948-66 gene cluster [45]. In this study, we confirmed the similarity of the gene content of strains carrying the *ptxP3 *allele in strains isolated in different countries. Moreover, we found that the *ptxP *allele is a good marker for strains that have similar gene content, since also the *ptxP2 *and *ptxP6 *strains are associated with the absence or presence of specific gene clusters (Figure 6). This indicates that strains with different *ptxP *types have different genetic backgrounds and form different lineages. Thus, these strains do not differ only in one point mutation, and therefore specific properties of these strains can also be related to this typical gene content. The *ptxP1 *strains are the most diverse in gene content although most gene clusters are typically lost in one strain or a specific group of strains. The *ptxP1 *strains missing BP0910-33, BP1135-41, BPP0511-2 and BPP2338-47 has the most single locus and double locus variants indicating that this is the older type. Strains with these characteristics are found in at least five different countries. The minimum spanning tree clearly showed that the *ptxP3 *clone emerged from the *ptxP1 *strain.

## Conclusions

In conclusion, an overview of genomic contents of a large collection of isolates from different countries allowed us to derive a core genome of *B. pertussis*. Clustering based on CGH analysis showed that strains isolated in different countries are highly similar in gene content. Furthermore, strains with the same *ptxP *type, exhibit great similarity in gene content suggesting that they form distinct lineages. Additionally, the CGH data revealed that the genome size of *B. pertussis *strains has decreased progressively over a period of 60 years. Our results show that *B. pertussis *is dynamic and is continuously evolving. This process seems to be influenced by adaptive evolution. Seemingly, there is selection for certain strains with smaller genomes. Although other factors may also influence the selection for these strains, these strains might benefit in the host with waning immunity by having lower cost of energy, with little negative effect on the host, or by removing gene products that are detrimental to the pathogenic lifestyle in older hosts.

## Methods

### Strains

*B. pertussis *isolates were isolated between 1949 and 2008 in six different countries; the Netherlands, Sweden, Australia, Kenya, Senegal and Japan. The strains selected for CGH analysis included the vaccine strains that were part of the Dutch WCV used before 2005. Other strains were selected based on frequency of occurrence of MLST types in each time period (see additional file 9, Table S8 for MLST frequencies). Thus, strains selected are representative of the most prevalent MLST-types in each time period. Swedish strains were selected based on Pulse Field type in accordance with frequency of occurrence. This work does not require approval of the ethical commission.

### Culture of strains and DNA isolation

The *B. pertussis *strains used for microarray analysis in this study are listed in additional file 3, Table S2. Strains were cultured for 72 hours on Bordet-Gengou agar plates at 35°C. Subsequently, they were grown at 35°C in Verweij medium (NVI, Bilthoven, Netherlands) with 200 μg ml^-1 ^heptakis (2, 6-di-o-methyl)-β-cyclodextrine for 24 hours while being shaken at 200 rpm. Chromosomal DNA was isolated using the Promega Wizard^®^Genomic DNA Purification Kit (Promega, Madison, USA), according to the manufacturer's instructions. The precipitated DNA was dissolved in 100 μl of EB (elution buffer, 10 mM Tris, pH 8.0) (Qiagen, Hilden, Germany).

### *B. pertussis *DNA array design and construction

The spotted 70-mer oligonucleotides microarray was constructed as described previously in [45]. Probes were spotted non-adjacent and in triplicate. The newly designed microarrays used in this study were custom-made pan-*Bordetella *microarrays using the 8 × 15 K format developed by Agilent Technologies, Wilmington, USA. The set of 5,910 60-mer oligonucleotides (60-mer), where one oligonulcleotide corresponds to one gene, covered 94% of the genes in the three sequenced *Bordetella *strains *B. pertussis *Tohama I, *B. parapertussis *12822 and *B. bronchiseptica *RB50 strain. *B. pertussis *oligonucleotides (60-mers) were deduced from the 70-mers used in the spotted microarray. In addition, 98 control probes were included in the microarray, and all spots were printed in duplicate (non adjacent). All user-defined probes were uploaded through the Agilent eArray Web portal (http://earray.chem.agilent.com/earray). All oligonucleotide sequences are listed (additional file 1, Table S1a and additional file 2, Table S1b). Additional details on the microarray production are available through the ArrayExpress microarray data repository (accession number A-MEXP-1695 for the spotted array and accession number A-MEXP-1697 for the extended Bordetella microarray).

### Labeling of genomic DNA and Microarray hybridization

For each CGH hybridization the DNA of a test strain and the reference strain ATCC BAA-589 Tohama I were labeled. Four micrograms of chromosomal DNA of *B. pertussis *were mixed with 20 μl of the 2.5 × Random Primer Mix (BioPrime DNA labeling kit; Invitrogen, Paisley, UK) in a total volume of 41 μl of water, boiled for 5 minutes, and then placed on ice. The samples were centrifuged for 2 minutes at 13,200 rpm and were mixed with 5 μl 10 × dNTP mix [2 mM dATP, dCTP and dGTP, 0.5 mM dTTP (Roche, Indianapolis, USA)], 2.5 μl of 1 mM Cy3 dUTP (for reference strain) or Cy5 dUTP (for the test strain) (GE Healthcare, Buckinghamshire, UK) and 1 μl Klenow polymerase (40 U μl ^-1^) (BioPrime DNA labeling kit; Invitrogen, Paisley, UK). The samples were incubated for 3 hours at 37°C. Subsequently, the test and reference samples were purified separately using CyScribe GFX Purification Kit (GE Healthcare, Buckinghamshire, UK) and then eluted in 60 μl elution buffer. The incorporation of the Cy-dyes in the labeled target sequences was measured with a NanoDrop spectrophotometer (NanoDrop Technologies).

For the spotted *B. pertussis *microarray in each CGH experiment, equal amounts of Cy3 dye (reference) and Cy5 dye (test) were used. The volume of the samples was decreased using a speedvac concentrator (New Brunswick Scientific, Edison, USA). The labeled test and reference samples were mixed together with 79.2 μl of hybridization solution [3.44 × SSC (Invitrogen, Paisley, UK), 0.32% SDS (Invitrogen, Paisley, UK), 1.0 mg yeast tRNA/ml]. Before loading on the microarray, the hybridization solution was heated for 3 minutes at 100°C and 5.8 μl of 10 × DIG blocking buffer was added.

For the Agilent pan-*Bordetella *microarray, 8 μl of Cy5 and 8 μl Cy3 labeled DNA were used if the specific activity was between 35-55 pmol/μg for Cy3 labeled DNA and between 25-40 pmol/μg for Cy5 labeled DNA. Cy5 and Cy3 labeled DNA were mixed with 4.5 μl Agilent 10 × blocking agent, 2 μl H_2_O and 22.5 μl Agilent 2 × hybridization buffer. Samples were further treated as described in Agilent protocols (Agilent oligonucleotide array-based CGH for genomic DNA analysis).

Spotted microarrays were hybridized and washed as previously described [45]. Agilent pan-*Bordetella *microarrays were hybridized and washed following Agilent's protocol (Agilent oligonucleotide array-based CGH for genomic DNA analysis).

### Microarray data mining

The hybridized slides were scanned at a 10 μm-resolution using a ScanArray Gx plus microarray scanner (Perkin Elmer) equipped with ScanArrray express software. Images from spotted microarrays were analyzed as previously described in [45]. The images from Agilent pan-*Bordetella *microarrays were analyzed using ImaGene software (Biodiscovery, El Segundo, USA). Individual arrays were internally normalized between the Cy3 and Cy5 channels by LOWESS normalization [58]. Based on hybridization results of ATCC BAA-589 Tohama I vs ATCC BAA-589 Tohama I, the cut-off for detection of a deletion was set at -1.5. Thus the normalized intensity ratio of < -1.5 indicates the absence of the gene. If the absence of these genes was not previously described, these genes were selected for further analysis by PCR or sequence analysis. The combined normalized data were visualized with TIGR MultiExperimentViewer. The logarithm of the hybridization ratio [log2 (Cy5/Cy3)] is indicated in the yellow-black-blue color scale. [log2 (Cy5/Cy3)]= -3 = Yellow, [log2 (Cy5/Cy3)] = 0 = Black, [log2 (Cy5/Cy3)] = +3 = Blue.

### PCR confirmation and sequence analysis

For strains analyzed with the spotted *B. pertussis *microarray, additional PCR confirmations were performed. Further PCR analysis was also employed in order to confirm the results predicted by the microarray hybridizations. The PCR primers were designed to target the flanking regions of each deletion so that the amplified region spanned the missing locus. The primers used in this study are listed in additional file 10, Table S9. The PCRs were performed under the following conditions: 20 μl total reaction volume, 10 μl Hotstart (Qiagen), 10 pmol of each primer (Eurogentec, Seraing, Belgium), 10 ng chromosomal DNA and 5% DMSO or 1.0 M Betaine (Sigma, St. Louis, USA). The amplification was carried out in a Geneamp PCR system 9700 thermocycler (Applied Biosystems, Foster City, USA) according to manufacturer's recommendations. After amplification, 10 μl of each PCR product was observed via 1% agarose gel electrophoresis with SYBR-Safe (Molecular Probes, Carlsbad, USA) staining. In order to determine the deletion boundaries, PCR products were purified using ExoSAP-IT^® ^(USB, Cleveland, USA). Subsequently the purified PCR products were sequenced using standard Big Dye Terminator v 3.1 (Applied Biosystems, Foster City, USA). Nucleotide sequencing was performed with an Applied Biosystems 3700 DNA Analyzer. Sequence data obtained from the ABI-3700 was compared to the *B. pertussis *sequence of Tohama I using the DNA-sequence analysis program KODON to determine the precise location of each deletion.

### Clustering and phylogenetic analysis

The final determination of absent (0) or present (1) was assigned to each RD for each strain in the CGH data and analyzed by BioNumerics version 5.1 (Applied-Maths, St. Maartens-Latem, Belgium). Clustering was carried out subsequently by the unweighted-pair group method using average linkages (UPGMA), to calculate a similarity matrix. A minimum spanning tree was built based upon the similarity matrix using Bionumerics version 5.1.

## Abbreviations

CGH: Comparative Genomic Hybridization; WCV: Whole Cell Vaccine; ACV: Acellular Vaccine; ISE: IS element

## Authors' contributions

AJK conceived the study, designed the experiments, carried out part of the experiments and data analysis and wrote the manuscript. TvG carried out part of the microarray experiments, PCR confirmations, DNA sequencing experiments. HGJvdH participated in microarray data analysis. AA supplied Swedish strains, carried out PFGE analysis and commented on the manuscript. SvdL carried out most of the microarray experiments, PCR confirmations, DNA sequencing experiments, part of the data analysis and was involved in writing the manuscript. All the authors read and approved the final manuscript.

## Supplementary Material

Additional file 1 Table S1a. Probes spotted on B. pertussis microarray Click here for file

Additional file 2 Table S1b. Probes printed on extended Bordetella microarray Click here for file

Additional file 3 Table S2. CGH analysis of B. pertussis strains used in this study Click here for file

Additional file 4 Table S3. Variable genes Click here for file

Additional file 5 Table S4. RDs Click here for file

Additional file 6 Table S5. Missing genes by others Click here for file

Additional file 7 Table S6. Core genes Click here for file

Additional file 8 Table S7. Color code COG domains Click here for file

Additional file 9 Table S8. MLST frequencies for Dutch (selected) strains Click here for file

Additional file 10 Table S9. Primers used in this study Click here for file
