# Supplementary material for: Changes in the genomic content of circulating Bordetella pertussis strains isolated from the Netherlands, Sweden, Japan and Australia: adaptive evolution or drift?
Source: BMC Genomics. 2010 Jan 26;11:64. doi: 10.1186/1471-2164-11-64 (PMC2834637; doi:10.1186/1471-2164-11-64)
Supplement: Additional file 8 — Table S7. Color code COG domains [file 1471-2164-11-64-S8.PDF]

- Translation, ribosomal structure and biogenesis
- RNA processing and modification
- Transcription
- Replication, recombination and repair
- Chromatin structure and dynamics
- Cell cycle control, cell division, chromosome partitioning
- Nuclear structure
- Defense mechanisms
- Signal transduction mechanisms
- Cell wall/membrane/envelope biogenesis
- Cell motility
- Cytoskeleton
- Extracellular structures
- Intracellular trafficking, secretion, and vesicular transport
- Posttranslational modification, protein turnover, chaperones
- Energy production and conversion
- Carbohydrate transport and metabolism
- Amino acid transport and metabolism
- Nucleotide transport and metabolism
- Coenzyme transport and metabolism
- Lipid transport and metabolism
- Inorganic ion transport and metabolism
- Secondary metabolites biosynthesis, transport and catabolism
- General function prediction only
- Function unknown
